# Documentation of Everyday Complaints in Veterinary Practice: Is There a Need for Greater Detail?

**DOI:** 10.3390/vetsci13060543

**Published:** 2026-05-30

**Authors:** Gabriel CuevasRamos

**Affiliations:** Large Animal Teaching Hospital, Copenhagen University, Agrovej 8, 2630 Taastrup, Denmark; gabriel.c.ramos@sund.ku.dk

**Keywords:** everyday complaints, veterinary practice, quality control

## Abstract

Everyday complaints (EDCs) in veterinary practice are frequently under-measured signals of quality and client experience. A practical framework to quantify and use these complaints is presented. It highlights that most published veterinary data describe formal legal or disciplinary cases, while routine concerns about fees, communication, outcomes, and service are rarely captured systematically and lack benchmarks linked to the workload of each practice. Drawing on veterinary mediation reports and human healthcare complaint taxonomies, the paper identifies key obstacles: unclear definitions, under-capture of informal complaints, absence of denominators, and failure to distinguish severity levels. To address this, the manuscript defines EDCs as any owner-initiated dissatisfaction requiring staff time (excluding legal/insurance cases) and outlines a form to log each episode with standardized fields such as category, channel, service area, severity, and resolution. It suggests veterinary clinics build internal baselines over at least 12 months, use run or control charts to detect meaningful shifts and treat both unexpectedly high and near-zero complaint rates as prompts for closer review, so veterinary hospitals can turn everyday complaints into structured inputs for quality improvement rather than purely reputational threats.

## Simple Summary

Everyday complaints in veterinary practice are often overlooked but can provide valuable insight into the quality of care and client experience. Most data focus on serious legal cases, while common issues like costs, communication, and service are rarely recorded in a structured way. This paper suggests a practical way to track and use these everyday complaints. It points out several challenges: there is no clear definition of what counts as a complaint, many informal complaints are not recorded, there is no way to relate complaints to the clinic’s workload, and the severity of complaints is often not distinguished. This approach defines everyday complaints as any owner’s dissatisfaction requiring staff time (excluding legal cases) and suggests recording them systematically. By tracking complaints over time and analyzing trends, clinics can use them as a tool to improve quality rather than just viewing them as problems.

## 1. Introduction

Everyday complaints (EDCs) in veterinary practice are common enough to affect staff wellbeing, client trust, and practice reputation, yet they are rarely captured with the same discipline used for clinical outcomes, safety incidents, or formal legal claims [1,2,3]. Even if there is published data, there is no clear method for assessing EDCs. How can we know when there is a real underlying problem? How relevant are they for veterinary practice workflow?

In the available veterinary literature, the best described complaints are lawsuits or disciplinary cases, but there is a lack of information regarding routine client concerns about fees, communication, perceived standards of care, unmet expectations, and service experience [1,3,4]. This creates a practical measurement problem for veterinary clinics and hospitals: complaints clearly matter, but there is no universally accepted benchmark expressed as complaints per 100 cases in veterinary medicine [1,3], nor is there a clear way to identify real, everyday unsatisfactory patterns, hence, how to possibly establish quality improvement programs.

The available data suggests that veterinary teams are exposed to complaints frequently, although most datasets measure staff experience or mediation referrals rather than EDC rates per consultation or per patient episode [1,3,5]. In one survey-based veterinary study, 34% of respondents reported receiving a client complaint within the previous 6 months, 51% stated complaints occurred every few months, and cost was the most frequently reported theme, cited by 79% of respondents [1]. UK mediation data also show that unresolved client concerns are numerous enough to generate thousands of annual enquiries, with dominant categories including standards of care, customer service, and fees [5,6,7]. Taken together, these findings indicate that everyday complaints are not rare outliers but part of routine practice operations, even if the exact denominator, prevalence, and how to approach them are missing [1,3]. We recognize that EDCs are part of the routine workflow of veterinarians, but we lack a specific framework on how to quantify them and, hence, improve our everyday practice.

Because specific veterinary rate studies are limited, human healthcare offers a useful methodological reference rather than a direct benchmark [8,9]. Large human hospitals often track complaints per 1000 patient encounters to normalize for caseload, and one academic medical center reported a yearly average of 1.27 complaints and grievances per 1000 patient encounters, while another hospital study reported 3.5 complaints per 1000 encounters [8,9]. These figures are not directly transferable to veterinary settings, but they show that complaint surveillance becomes much more meaningful once a clinic defines a standard denominator and monitors trends over time rather than relying on anecdotal reports [8,9,10].

## 2. Why Is There a Lack of Information on EDCs in Veterinary Clinics?

The first difficulty is definitional. Many practices do not distinguish between a complaint, a criticism, a billing query, a refund request, a negative online review, and a safety-related grievance, even though these events have different meanings and different quality improvement implications [3,11]. If a client says that the bill was unexpected, that may represent a communication failure, a financial consent failure, or simply dissatisfaction despite appropriate prior explanation [4,7]. Without agreed definitions, complaint counts are unstable and comparisons between clinicians, departments, or months (seasonal) become unreliable [3,8].

The second difficulty is under-capture. Many complaints are managed informally at reception, by phone, by email, or during discharge, and are resolved before they ever enter a formal complaint log [3,11]. Veterinary mediation organizations explicitly describe their role as dealing with complaints that could not be resolved locally within the practice, which means external mediation represents only the visible tail of a much larger internal complaint burden [3,5]. In other words, a clinic with very few formal complaints may not truly have few complaints; it may simply have weak recording systems or a culture that absorbs concerns without classifying them [3,8].

A third difficulty is the absence of a valid denominator. Counting 20 complaints in one month is uninterpretable unless it is related to activity, such as 400 consultations, 120 procedures, 85 inpatients, or 300 invoices [8,9]. A referral hospital, emergency service, and first-opinion practice have very different case mixes and client expectations, so raw totals can mislead [9,10]. Complaint surveillance therefore must move away from absolute numbers and toward rates, stratified by service type and tracked longitudinally [8,10].

A fourth difficulty is that complaint severity varies widely. A short call about a confusing invoice should not carry the same analytic weight as a prolonged dispute about communication around a poor outcome [3,8]. Human healthcare complaint systems often add severity scoring and category coding because a stable low-level background of minor complaints may be operationally acceptable, while a smaller number of high-severity complaints may indicate serious system risk [8,10]. Veterinary practices could benefit from adopting the same logic [3,11].

## 3. How to Measure EDCs?

A practical veterinary framework should begin with a broad operational definition: an everyday complaint is any owner-initiated expression of dissatisfaction requiring staff time beyond routine clarification, excluding claims already escalated to insurers, lawyers, or disciplinary authorities [3,11]. This definition should include in-person complaints, telephone complaints, email or written complaints, unresolved billing objections, and complaints embedded in online messages when the practice engages with them [3,11]. The goal is not to label all unhappy communication as misconduct, but to create a reproducible dataset [8].

Each complaint should be logged once as a complaint episode, even if it generates multiple contacts, and then coded across several fields [8,9]. The minimum fields should include date, service area, attending clinician or team, complaint channel, whether the case involved emergency care, a refund or discount was requested, and primary complaint category [3,8]. Categories should be simple and stable, such as fees/billing, communication, access and waiting, service behavior, perceived outcome, perceived clinical care, and administrative errors [1,8,10].

Measurement becomes useful when the clinic chooses one primary denominator and one or two secondary denominators. The most defensible primary rate is complaints per 1000 clinical encounters, because it captures the number of client-facing episodes and allows comparison across months and sites [8,9]. Secondary rates may include complaints per 100 invoices, which is helpful for fee-related complaints, and complaints per 100 procedures or admissions, which may be more relevant in referral hospitals where outcomes and consent discussions are more complex [7,9].

A simple reporting dashboard (Table 1) can therefore include the following indicators:

– Total complaint episodes per month [8].
– Complaints per 1000 encounters [8,9] (determined according to the workload of the practice).
– Complaints per 100 invoices for billing-related issues [4,7] (determined according to the workload of the practice).
– Proportion by category; for example, fees, communication, standards of care, and service [1,6,8].
– Time to first response and time to closure, because responsiveness influences whether concerns escalate [3,5].
– Proportion resolved locally versus escalated externally [3,5].
– Severity score, such as 1 to 3 or 1 to 5, to distinguish inconvenience from major trust breakdown [8,10].

To improve reliability, clinics should also track the source of complaint [8,11]. A complaint entered by reception after a phone call is different from one identified through a post-visit survey and shifts in reporting channels can change rates even when the true underlying dissatisfaction has not changed [8]. This is one reason why complaint data should be interpreted alongside patient or client experience surveys rather than in isolation [10,12].

## 4. How to Judge Whether There Are Too Many or Too Few EDCs

In the current veterinary literature, no robust external benchmark defines a normal threshold or expected number of EDCs per 100 cases for all practice types [1,12]. For that reason, a clinic should resist using a single universal threshold and instead build an internal baseline over at least 12 months, ideally stratified by service line such as first opinion, emergency, surgery, medicine, and hospitalization [8,9]. The central question is less “What is the perfect number?” and more “Is the clinic stable, improving, or deteriorating after adjustment for workload and services” [8,10].

A reasonable interpretive approach is to combine statistical surveillance with managerial judgment. First, calculate the monthly complaint rate per 1000 encounters and the category-specific rates, then display them as run charts or statistical process control charts. A sustained shift above the clinic’s own median, repeated points above the upper control limit, or a sudden rise in one category, such as billing or outcome, should be treated as a signal that complaints are above expected variation [8,10].

Second, define escalation triggers based on both frequency and severity. For example, a clinic may decide that a month with a stable total complaint rate, but a doubling of high-severity outcome or communication complaints still represents excessive complaint burden because the risk to trust and reputation is greater. Conversely, very low complaint rates may not always be reassuring; if surveys, online reviews, staff reports, or unpaid invoices suggest friction, a near-zero complaint log may indicate under-reporting rather than exceptional performance [3,8].

Human healthcare offers a useful perspective here. Published hospital complaint rates of roughly 1.27 to 3.5 per 1000 encounters demonstrate that even highly organized medical systems expect some background level of dissatisfaction, particularly around communication and perceived care quality [8,9]. Veterinary clinics should therefore be cautious about aiming for a “low level” of complaints and should instead aim for rapid capture, fair resolution, low recurrence, low severity, and realistic expectations [3,11].

## 5. Possible Solutions for Veterinary Clinics and Hospitals

The first solution is to create a standardized complaint taxonomy and train all front-desk, nursing, and veterinary staff to use it consistently. The taxonomy should be intentionally small so that routine logging is feasible but detailed enough to separate financial-consent problems from clinical-outcome dissatisfaction and interpersonal communication failures. Standardization converts complaint handling from anecdotal information into a quality dataset [1,8,11].

The second solution is to embed complaint capture into a routine workflow. A digital form linked to the practice management system can allow any staff member to log a complaint in under two minutes, with mandatory fields for category, severity, and whether the complaint was resolved at first contact (Table 1). If logging is cumbersome, the clinic will systematically underestimate complaint burden [3,8,11].

The third solution is to pair complaint surveillance with denominator discipline and regular review. Monthly dashboards should be reviewed by leadership and by service heads, not merely filed away, and should present both counts and rates. Segmenting the data often reveals actionable patterns, such as billing complaints clustering after emergency admissions, or communication complaints increasing on weekends, at discharge, or after clinician handovers [8,9,10].

The fourth solution is to treat complaints as leading indicators, not only as reputational threats. In human healthcare, complaint analysis has been used to detect communication problems, process failures, and perceived safety concerns that conventional incident reporting may miss [8,10]. The same logic is highly relevant to veterinary care, where outcome uncertainty, emotional decision-making, and price sensitivity can amplify dissatisfaction before any formal legal action occurs [2,3].

The fifth solution is better financial communication. Veterinary surveys and mediation reports repeatedly identify cost or fee concerns as a major driver of complaints, which means transparent estimates, documented consent discussions, and early updates on budget changes are not only customer-service practices but also complaint-prevention tools [1,4,7]. Where possible, clinics should audit complaints against estimate accuracy, invoice revisions, and whether major cost discussions were documented before treatment proceeded [4,7].

Finally, clinics should distinguish between signal and noise, seasonal (end of year) versus recurrent. A moderate number of minor complaints may be compatible with a busy, transparent service, whereas repeated complaints about the same workflow, same discharge process, or same pathway indicate a system problem that deserves intervention [8,10]. The most mature complaint systems therefore ask not only “How many complaints occurred?” but also “How often do the same causes recur? How severe are they? And are corrective actions reducing recurrence over time?”.

## 6. Perspective

The absence of strong published veterinary benchmarks does not make everyday complaints unmeasurable. It means that clinics must borrow proven measurement principles from healthcare quality science, especially the use of clear definitions, standardized coding, encounter-based denominators, severity grading, and longitudinal trend analysis. With this approach, a veterinary hospital will be able to determine not only how many everyday complaints it receives, but also whether the observed level is truly excessive, merely expected, or low because the system is not capturing what clients are already expressing.

## Figures and Tables

**Table 1:** Every Day Complaint Recording Form.

SECTION	FIELD	ENTRY/OPTIONS
**A. BASIC INFO**	Complaint ID	e.g., 2026-EDC-001
	Date and time of complaint	_/_/(DD/MM/YYYY) _:_ (HH:MM)
	Linked client	Client name/ID:
	Linked patient	Patient name/ID: _
	Linked invoice/visit	Invoice/visit number: _
**B. CONTEXT**	Service area	☐ First opinion consult ☐ Referral ☐ Emergency ☐ Surgery ☐ Hospitalization ☐ Imaging ☐ inpatient care ☐ Admin/Other:
	Case type	☐ New case ☐ Follow-up ☐ Control ☐ Repeat prescription ☐ Administrative only
	Species	☐ Dog ☐ Cat ☐ Horse ☐ Other:
**C. CLIENT & CHANNEL**	Contact role	☐ Owner ☐ Co-owner ☐ Referring vet. ☐ Other:
	Channel (primary)	☐ In person ☐ Phone ☐ Email ☐ Letter ☐ Online review/social media ☐ Web form ☐ Other:
	Who first received complaint?	☐ Reception ☐ Nurse ☐ Veterinarian ☐ Manager ☐ Other:
**D. COMPLAINT CONTENT**	Primary category (tick ONE)	☐ Fees/billing/estimates ☐ Communication/information ☐ Perceived clinical care/standards ☐ Perceived outcome/prognosis ☐ Access/waiting/availability ☐ Staff behaviour/attitude ☐ Administration/scheduling/documentation ☐ Facilities/environment ☐ Other:
	Emergency case?	☐ Yes ☐ No
	Estimate provided?	☐ Yes–written ☐ Yes–verbal ☐ No ☐ Unknown
	Invoice disputed?	☐ Yes ☐ No
	Refund/discount requested?	☐ Yes ☐ No
	Refund/discount given?	☐ Yes–full ☐ Yes–partial ☐ No
**E. SEVERITY & NARRATIVE**	Severity score (overall)	Circle one: 1 = Minor 2 = Moderate 3 = Major 4 = High risk (threat of board/insurer/legal/online escalation)
	Risk to patient safety?	☐ None ☐ Possible ☐ Clear safety concern
**F. RESOLUTION**	Resolved at first contact?	☐ Yes ☐ No
	Staff member responsible for resolution	Name/initials/role:
	Actions taken (tick all that apply)	☐ Explanation/apology ☐ Additional information/follow-up call ☐ Clinical re-check offered ☐ Fee adjustment/refund ☐ Written response sent ☐ Policy/process change initiated ☐ Training/feedback to staff ☐ Other:
	Date closed	_/_/(DD/MM/YYYY)
	Outcome (internal)	☐ Fully resolved, client satisfied ☐ Resolved, client still somewhat dissatisfied ☐ Not resolved/client left practice ☐ Unknown (no further contact)

## Data Availability

The original contributions presented in this study are included in the article. Further inquiries can be directed to the corresponding author.
